# Residents learning ultrasound-guided catheterization are not sufficiently skilled to use landmarks

**DOI:** 10.1186/cc13317

**Published:** 2014-03-17

**Authors:** J Maizel, L Guyomarc'h, P Henon, S Samy Modeliar, B De Cagny, G Choukroun, M Slama

**Affiliations:** 1 CHU Amiens, France

## Introduction

An ultrasound-guided (UG) technique is the recommended procedure for central venous catheterization (CVC). But ultrasound may not be available in emergency situations, and therefore guidelines also propose that physicians remained skilled in landmark (LM) placement. We conducted this prospective observational study to determine the learning curve of the LM technique in residents only learning the UG technique.

## Methods

During the first 3 months of their rotation in our ICU, residents inexperienced in CVC used only the real-time UG technique. During the following 3 months, residents were allowed to place CVC by means of the LM technique when authorized by the attending physician.

## Results

A total of 172 procedures (84 UG and 88 LM) were performed by the inexperienced residents during the study. The success rate was lower (72% vs. 84%; *P = *0.05) and the complication rate was higher (22% vs. 10%; *P = *0.04) for LM compared with UG procedures. Comparison between the five last UG procedures and the first five LM procedures performed demonstrated that the transition between the two techniques was associated with a marked decrease of the success rate (65% vs. 93%; *P = *0.01) and an increase of the complication rate (33% vs. 8%; *P = *0.01). After 10 LM procedures, residents achieved a success rate and a complication rate of 81% and 6%, respectively.

## Conclusion

Residents who only learn the UG technique will not be immediately able to perform the LM technique, but require specific training based on at least 10 LM procedures. Whether the LM technique should still be taught when an ultrasound device is not available must therefore be addressed.

